# Exploring the Presenting Problems and Services Received by Youth with Diverse Mental Health Presentations in Integrated Youth Mental Health Services Across Canada

**DOI:** 10.5334/ijic.10147

**Published:** 2026-03-18

**Authors:** Sarah Mughal, Alba Sanchez-Allakhverdieva, Amal Abdel-Baki, Neil Andersson, Patricia Boksa, Daphne Hutt-Macleod, Ridha Joober, Shalini Lal, Jessica Chisholm-Nelson, Nora Morrison, Alyssa Frampton, Mary Anne Levasseur, Lacey Augustine, Kevin Friese, Isabelle Godin, Katherine Hay, Norma Rabbitskin, Paula Reaume-Zimmer, Helen Vallianatos, Srividya N. Iyer, Ashok Malla, Jai L. Shah, On behalf of ACCESS Open Minds

**Affiliations:** 1 Department of Psychiatry, McGill University, Montreal, Quebec, Canada; 2 Douglas Mental Health University Institute, Montreal, Quebec, Canada; 3 ACCESS Open Minds, Canada; 4 Centre de recherche du Centre hospitalier de l’Université de Montréal, Montréal, Québec, Canada; 5 Dans La Rue and Réseau d’intervention de proximitéauprès des jeunes de la rue-Montréal/Homeless Youth Network, Montréal, Québec, Canada; 6 Department of Psychiatry, Université de Montréal, Montréal, Québec, Canada; 7 Centre hospitalier de l’Université de Montréal, Montréal, Québec, Canada; 8 Department of Family Medicine, Community Information and Epidemiological Technologies Institute and Participatory Research at McGill (PRAM), McGill University, Montréal, Québec, Canada; 9 McGill University Institute for Human Development and Well-being, Montréal, Québec, Canada; 10 ACCESS Open Minds Eskasoni First Nation, Eskasoni First Nation, Nova Scotia, Canada*; 11 Now with Nova Scotia Integrated Youth Services Initiative, Nova Scotia, Canada; 12 Prevention and Early Intervention Program for Psychosis, Douglas Mental Health University Institute, Montreal, Quebec, Canada; 13 School of Rehabilitation, Faculty of Medicine, Université de Montreal, Montreal, Quebec, Canada; 14 Now with Faculty of Medicine and Health, University of Sydney, Sydney, NSW, Australia; 15 Department of Epidemiology, Biostatistics and Occupational Health, McGill University, Montréal, Québec, Canada; 16 ACCESS Open Minds Youth Council, Douglas Mental Health University Institute, Montreal, Quebec, Canada; 17 ACCESS Open Minds Family and Carers Council, Douglas Mental Health University Institute, Montreal, Quebec, Canada; 18 Elsipogtog Health and Wellness Centre and ACCESS Open Minds New Brunswick*, Elsipogtog First Nation, New Brunswick, Canada; 19 ACCESS Open Minds University of Alberta, Edmonton, Alberta, Canada*; 20 Office of the Dean of Students, University of Alberta, Edmonton, Alberta, Canada; 21 ACCESS Open Minds-Esprits ouverts New Brunswick, Acadian Peninsula, Moncton, New Brunswick, Canada*; 22 Centre de Bénévolat de la Péninsule Acadienne, New Brunswick, Canada; 23 Now with Kickstand Integrated Youth Services Initiative, Alberta, Canada; 24 ACCESS Open Minds Edmonton, Edmonton, Alberta, Canada*; 25 Now with Big River Health Centre, Saskatchewan, Canada; 26 Sturgeon Lake Health Centre, Sturgeon Lake First Nation, Saskatchewan, Canada*; 27 ACCESS Open Minds Sturgeon Lake First Nation, Saskatchewan, Canada*; 28 Now with Bluewater Health, Sarnia, Ontario, Canada; 29 ACCESS Open Minds Chatham-Kent, Chatham-Kent, Ontario, Canada*; 30 Department of Anthropology, University of Alberta, Edmonton, Alberta, Canada

**Keywords:** youth mental health, integrated youth services, accessibility, severity, health equity

## Abstract

**Background:**

Integrated youth mental health (YMH) primary care initiatives have the potential to improve Canada’s treatment gap. However, there is no current standard for implementing these services, and offerings vary greatly across models and sites. This has equity implications for the care received by youth with severe presentations.

**Methods:**

We describe the provider-rated problems with which 2995 youth presented to ACCESS Open Minds, a multi-site, Canadian YMH integrated care initiative and the services they received, based on the severity of their needs at intake.

**Results:**

The largest proportion of youth had both moderate-severe presentations and moderate-to-significant difficulties in functioning (41.67%). The first instance of receiving services following intake for most participants (71.42%) entailed a single service. The highest proportion of youth for whom the type of service received was not documented was those who presented with neither moderate-severe presentations nor poor functioning (15.01%). There was substantial variability in services provided across sites.

**Conclusion:**

Variability in service delivery confirms the need for greater standardization of core service offerings across sites. Future research should assess the appropriateness of services for different presentations to ensure equitable support is provided for all youth.

## Introduction

Young people in Canada need timely and high-quality mental health services. Both self-reported mental health problems and diagnosed anxiety and mood disorders increased among Canadian youth from 2011–2018 [1], as have past-year mental health consultations which rose from 11.7% in 2011 to 17.0% in 2018 [1]. Yet, despite increased demand for care, 41% of Canadian adolescents are still not receiving adequate mental health treatment [2]. This gap is related to multiple issues including complex care pathways, growing waitlists, service and systems silos, poor coordination, and treatment deserts where no appropriate services are available [3,4].

Youth mental health (YMH) issues require diverse interventions tailored to severity and need. Evidence suggests the treatment gap may be wider for young people with more severe or complex mental health problems who have been shown to receive lower quality care [5], more disjointed care [6], greater barriers to accessing care [7], or a lack of appropriate treatments altogether [8]. This confirms a need for system reforms aimed at improving the mental health of young people – particularly those with more severe or complex concerns.

Emerging initiatives aim to reduce the youth treatment gap. Enhanced primary YMH care, including Integrated Youth Services (“IYS”), is gaining traction across Canada and other high-income countries [9,10], with at least 12 countries implementing some form of IYS as of 2022 [11]. The contemporary IYS concept is as an integrated model aimed at enhancing primary care by co-locating and coordinating a variety of mental, physical, and social services for youth. The intention is to create a transdiagnostic “one-stop-shop”, where all of a young person’s needs can be met in one place [12,13]. However, some question whether IYS can adequately address the needs of youth with severe clinical presentations [14].

There are no agreed standards for operationalizing or integrating enhanced primary YMH care initiatives [15]. As a result, implementation varies significantly and different clinical and non-clinical services are offered from site to site [11,16]. This may lead to varying degrees of success in addressing the diverse mental health problems of youth seeking care [12,14,17].

Efforts to evaluate enhanced primary YMH initiatives have yielded conflicting results regarding clinical impacts [12,18]. It has been suggested that individuals who receive fewer treatment sessions and/or those with the most severe presentations may fail to experience reductions in clinical symptoms [12]. ACCESS Open Minds (AOM), a multi-site Canadian service transformation initiative, reported that youth with moderate-severe presenting concerns experienced longer wait times for initial appointments and services [19]. These findings are notable given that an estimated 69–77% of young people who seek support in many primary YMH care settings have high and/or severe levels of need [12,20,21].

If young people with more severe presentations do not benefit equally from enhanced primary YMH care initiatives in their current iterations, research to understand how different models assess and support varying YMH needs is critical. This underscores the imperative to better understand a) which mental health needs are being met in these settings, and b) how various sites meet varied needs with the services they provide, with particular attention on potential gaps for severe presentations.

Our study describes the problems young people presented with at AOM, and the services they received, stratified by the severity of their mental health needs at intake. We also examine site-specific patterns in presenting problems and services received, given potential site-level differences. The goal of this work is to guide improvements in this Canadian, extended primary YMH care model to better meet the needs of all young people who access these novel service hubs.

## Methods

### Setting & Sample

AOM is a pan-Canadian youth mental health service transformation project, taking place in 14 sites in five provinces and one territory [22]; including six in Indigenous communities [23], three in rural and semi-urban areas [22], and five in urban centres [3]. Among urban sites, two were special population sites – one being university-based [24], and another that supported homeless youth [25]. AOM began planning in 2013, started operations in 2016, and concluded its data collection in 2020 [3]. Site locations reflected diverse geographic, linguistic, and sociodemographic contexts. The intervention package was intended to provide an integrated alternative to pre-existing mental health services for young people aged 11–25 (regardless of stage or presence of a diagnosis), and to generate evidence regarding its impact on individual and systemic outcomes [3,22]. Sites differed in services provided and delivery methods to align with local needs, but all shared common objectives including early case identification, rapid access, youth/family engagement, and eliminating age-based transitions [19].

The complete dataset used for these analyses includes data from 5205 participants across 10 participating sites (site sample sizes ranging from 64 to 1562 youth). Two Inuit sites did not participate in the evaluation protocol. Two First Nations sites were not included due to incomplete data on the variables of interest or in keeping with wishes of the community.

### Ethics

Ethics approval was obtained from the Research Ethics Board of the Douglas Mental Health University Institute (IUSMD-15-21), and local institutional, community, and First Nations bodies relevant for each site. This study was conducted following Ownership, Control, Access, and Possession (OCAP™) principles as well as Tri-Council guidelines for research involving Indigenous Peoples [26].

### Measures

The AOM intervention was evaluated across all sites via a common, minimum evaluation protocol [3]. Data collected as part of this effort includes sociodemographic data (e.g. age, gender), service implementation measures (e.g. portals of entry), individual outcomes (i.e. psychological distress, social and occupational functioning), and system-level outcomes (i.e. delay between assessment and access to care).

For this study, we used the following data:

1. **AOM Site:** 10 of the 14 total sites were included (presented alphabetically): Acadian Peninsula (NB), Chatham-Kent (ON), Dorval-Lachine-Lasalle (QC), Edmonton (AB), Elsipogtog First Nation (NB), Eskasoni First Nation (NS), Parc-Extension (QC), PEER Saint John (NB), RIPAJ-Montréal (QC), and University of Alberta (AB).
2. **Socio-demographic Variables:** The sociodemographic characteristics described in this analysis were gender, mean age, visible minority (yes/no), Indigenous identity (yes/no), sexual orientation, and living situation. In AOM’s demographic intake survey, the “sexual orientation” options included: asexual, bisexual, gay, heterosexual/straight, lesbian, queer, questioning, not sure, prefer not to answer, and other. For this analysis, responses were grouped as either straight/heterosexual or LGBQ+ which includes all other possible responses. Living situation options included: house, dormitory/residence, single room in someone else’s house, apartment, group home, supported housing, homeless shelter, on the street, couch surf, and other. For this analysis, the first four options were grouped as “stable housing” and the rest as “precarious housing”.
3. **Presenting Problems:** Providers identified presenting problems on intake from a checklist (built with inputs from various stakeholder groups across sites) of 67 concerns across the following categories: cognition, physical health, emotional/psychological concerns, context/lifestyle, behavioural concerns (including both self-injury and substance use concerns), identity/sexuality, school/work/occupational concerns, family relationships, peer relationships/social interactions, community/living situation, trauma/abuse-related issues, and other. Providers could select all that applied for each young person. Presenting problems are not diagnostic categories and should not be assumed to reflect diagnoses.
4. **Intake Severity Categories:** Scores from provider-rated Clinical Global Impressions (CGI) and Social and Occupational Functioning Assessment Scale (SOFAS) intake assessments were used to make a composite severity variable. This multidimensional composite measure was created in order to generate three severity categories inclusive of both youth distress and functional impairment. First, each score was independently categorized as “moderate-severe” if CGI ≥ 4 [27], and “moderate-to-significant difficulties in functioning” if the SOFAS ≤ 60 [28]. Stratification for these analyses were then conducted using the following categories:
  1. “Moderate-Severe and Poor Functioning” if a participant meets moderate-severe and moderate-significant difficulties in functioning criteria on both scales (i.e. CGI ≥ 4 and SOFAS ≤ 60);
  2. “Moderate-Severe or Poor Functioning” if a participant meets the moderate-severe and moderate-significant difficulties in functioning criteria on either one of the two scales;
  3. “Not Moderate-Severe and Not Poor Functioning” if a participant meets the moderate- severe and moderate-significant difficulties in functioning criteria on neither scale.
5. **First Service Offering Type and Number of First Services Documented:** Following intake, trained staff recorded date of referral, date of first mental health evaluation, date of first service(s) received, and the type(s) of first services received. Service types were categorized into 22 service types based on *a priori* list and coding of open-ended responses (Supp Table 6). In this record, “first service(s)” include all documented interventions provided on the earliest recorded date to the young person following their initial intake. Importantly, young people may have received additional (undocumented) services following this initial record; these are not included in this variable.

### Data Analyses

We first describe the complete AOM dataset with frequencies and proportions of sociodemographic characteristics, CGI, and SOFAS scores stratified by AOM site. The analytic sample for subsequent analyses is the subsample of participants with complete CGI and SOFAS data. It also excludes youth who did not receive a service beyond the initial evaluation; those who sought services after February 1 2020; and youth who had data missing on time of referral/services received. This was to consolidate data with severity variables of interest and to remove individuals whose service pathways were affected by the COVID-19 pandemic.

In the analytic sample, we describe the frequency and percentages of all three intake severity categories, stratified by AOM site. We then describe the proportions of provider-rated presenting problems, the proportions of participants who received 1, 2, or 3+ first services on the earliest recorded date following intake or for whom type/number of services was not documented, and the proportions of individuals who received each service type, stratified by intake severity category and by AOM site. Service type received is also stratified by presenting problem on intake.

### Lived Experience

A foundational principle of AOM is that this project be designed, implemented, and evaluated with the involvement of diverse lived experience stakeholder groups, inclusive of both youth and family members of youth with lived experience. Decisions about AOM core values, minimum evaluation protocol, and intake assessment tools used in these analyses each involved lived experience stakeholders via cross-network and site-specific advisory councils. In addition, multiple authors of this manuscript identify as having lived experience.

## Results

### Sample Characteristics – Complete vs. Analytic Samples

#### Complete Sample

The complete sample includes 5205 participants who received an initial evaluation at one of the 10 participating AOM sites and consented to this study. Two sites comprised the majority of the dataset (54.8%), and four sites each represented <5% of the sample. The mean age of participants was 19.24 years and 50.43% were female. Additionally, 17.89% of participants identified as Indigenous and 12.37% experienced housing insecurity on intake. Notable differences emerged across sites: the site dedicated to homeless youth had a particularly high proportion of insecurely housed participants (63.29%), and the two First Nations on-reserve sites exclusively supported First Nations participants from their communities (100%). Missing data varied across sociodemographic variables and sites, ranging from 0% to 80.76% for some variables (Supp Table 1). 46.63% of young people had moderate-severe presentations on the CGI and 37.29% had moderate-to-significant difficulties in functioning on the SOFAS (Supp Table 2). Across sites, 21.59% of participants had missing CGI data and 26.05% had missing SOFAS data (Supp Table 2).

#### Analytic Sample

The analytic sample included 2995 participants. The same two sites as in the complete sample comprised the majority of the dataset (53.7%), and the same four sites as in the complete sample each represented <5% of the sample. The average age in the analytic dataset was 19.47, 51.52% were female, 13.89% participants were Indigenous, and 13.66% experienced housing insecurity on intake. The same site dedicated to homeless youth had the highest proportion of participants with precarious housing (70.08%), and the same two on–reserve sites served exclusively First Nations populations. Missing data on sociodemographic variables was lower in the analytic sample, ranging from 0% to 68.17% (Supp Table 3). 65.34% had moderate-severe presentations on the CGI and 49.84% had moderate-to-significant difficulties in functioning on the SOFAS (Table 1).

**Table 1:** CGI^a^ and SOFAS^b^ in the Analytic Sample.


SITE	N	CGI SEVERITY				SOFAS SEVERITY			
	
		MILD		MODERATE-SEVERE		NO-LOW DIFFICULTIES IN FUNCTIONING		MODERATE-SIGNIFICANT DIFFICULTIES IN FUNCTIONING	

**1**	**281**	55	19.57%	226	80.43%	125	44.48%	156	55.52%

**2**	**192**	75	39.06%	117	60.94%	93	48.44%	99	51.56%

**3**	**508**	119	23.43%	389	76.57%	35	6.89%	473	93.11%

**4**	**600**	272	45.33%	328	54.67%	293	48.83%	307	51.17%

**5**	**109**	57	52.29%	52	47.71%	82	75.23%	27	24.77%

**6**	**1009**	284	28.15%	725	71.85%	663	65.71%	346	34.29%

**7**	**137**	83	60.58%	54	39.42%	102	74.45%	35	25.55%

**8**	**59**	24	40.68%	35	59.32%	30	50.85%	29	49.15%

**9**	**86**	62	72.09%	24	27.91%	73	84.88%	13	15.12%

**10**	**14**	7	50.00%	7	50.00%	6	42.86%	8	57.14%

**Total**	**2995**	1038	34.66%	1957	65.34%	1502	50.15%	1493	49.84%


^a^Clinical Global Impressions. ^b^Social and Occupational Functioning.

### Analyses in Analytic Sample

#### Intake Severity Categories

Across severity categories, the largest proportion (41.67%) of participants had both moderate-severe presentations and moderate-to-significant difficulties in functioning. In comparison, 31.85% presented as either moderate-severe or poor functioning, and 26.48% presented as not moderate-severe and not poor functioning. The proportion of young people presenting as both moderate-severe and poor functioning ranged from 10.74% to 75.79% across sites (Table 2).

**Table 2:** Intake Severity Categories By Site.


SITE	N	SEVERITY MEASURES					

		NOT SEVERE; NOT MODERATE-TO-SIGNIFICANT FUNCTIONING DIFFICULTIES		EITHER SEVERE OR MODERATE-TO-SIGNIFICANT FUNCTIONING DIFFICULTIES		BOTH SEVERE & MODERATE-TO-SIGNIFICANT FUNCTIONING DIFFICULTIES	

**1**	**281**	45	16.01%	90	32.03%	146	51.96%

**2**	**192**	59	30.73%	50	26.04%	83	43.23%

**3**	**508**	31	6.10%	92	18.11%	385	75.79%

**4**	**600**	199	33.17%	167	27.83%	234	39.00%

**5**	**109**	50	45.87%	39	35.78%	20	18.35%

**6**	**1009**	249	24.68%	449	44.50%	311	30.82%

**7**	**137**	77	56.20%	31	22.63%	29	21.17%

**8**	**59**	20	33.90%	14	23.73%	25	42.37%

**9**	**86**	58	67.44%	19	22.09%	9	10.47%

**10**	**14**	5	35.71%	3	21.43%	6	42.86%

**Total**	**2995**	793	26.48%	954	31.85%	1248	41.67%


### Presenting Problems

#### By Intake Severity Category

The most frequently documented clinician-rated presenting problems across severity categories were anxiety/worry (42.70%), depression/sadness (29.98%), and stress (29.62%). Apart from these, the issues most frequently documented for individuals presenting as both moderate-severe and poor functioning were financial instability (26.68%), substance misuse (self) (24.36%), loneliness/isolation (23.24%) and suicidal thoughts (20.99%). The additional issue most frequently documented for individuals presenting as not moderate-severe and not poor functioning was concentration difficulties (15.29%) (Supp Table 4).

#### By Site

Anxiety/worry, stress, and depression/sadness were the most frequent presenting problems across most sites. Presenting problems at the two ‘special population sites’ for homeless youth and for post-secondary youth) were consistently found to be in keeping with the characterization of those sites (homelessness and financial instability in the site supporting homeless youth, and higher levels of academic difficulties at university-based site) (Supp Table 5).

### Documented Number of First Services Received

#### By Intake Severity Category

The first instance of receiving services following intake for most participants (71.42%) entailed a single service. This was true across all three severity categories (Table 3). The highest proportion of young people for whom the number of services was not documented was for those who presented as not moderate-severe and not poor functioning (15.01%), followed by those who presented as both moderate-severe and poor functioning (13.14%), and as either moderate-severe or poor functioning (11.64%). The highest proportion of young people to receive either 2 or 3+ services were those who presented as not moderate-severe and not poor functioning (3.91%) (Table 3).

**Table 3:** Number of Services Received by Severity.


NUMBER OF SERVICES RECEIVED	TOTAL		SEVERITY MEASURES					
	
	(N = 2995)		NOT SEVERE;NOT MODERATE-TO-SIGNIFICANT FUNCTIONING DIFFICULTIES (N = 793)		EITHER SEVERE OR MODERATE-TO-SIGNIFICANT FUNCTIONING DIFFICULTIES(N = 954)		BOTH SEVERE & MODERATE-TO-SIGNIFICANT FUNCTIONING DIFFICULTIES (N = 1248)	

**Zero/None Documented**	394	13.16%	119	15.01%	111	11.64%	164	13.14%

**One Service**	2139	71.42%	533	67.21%	693	72.63%	913	73.16%

**Two Services**	380	12.69%	110	13.87%	126	13.21%	144	11.54%

**Three+ Services**	82	2.71%	31	3.91%	24	2.52%	27	2.16%


#### By Site

There was significant variation across sites in the proportion of participants for whom the number of services was not documented (from 2.38% to 42.86%) (Supp Table 6). Few participants received 3+ documented services in the first instance, with one site being an exception (Supp Table 6).

### Service Type

#### By Intake Severity Category

Individual Therapy (26.04%) and Single Session Therapy with Care Coordination (22.54%) were the most common first services received. The proportion of young people receiving other service options did not appear to vary based on severity category. For example, crisis intervention was received by 0.13–1.28% of young people across severity categories. One exception was for those who received services from a mental health specialist or received a service package. This was the first service received by 5.53% of young people who presented as both moderate-severe and poor functioning, but was the first service received by less than 1% of young people in the other severity categories. Conversely, case management was most frequently the first service received by youth who presented as not moderate-severe and not poor functioning (21.44%), compared to youth who presented as both moderate-severe and poor functioning (17.84) (Table 4).

**Table 4:** Service Type by Severity.


SERVICE RECEIVED -TYPE	TOTAL		COMPOSITE SEVERITY MEASURE					
	
	(N = 2995)		NOT SEVERE; NOT MODERATE-TO-SIGNIFICANT FUNCTIONING DIFFICULTIES (N = 793)		EITHER SEVERE OR MODERATE-TO-SIGNIFICANT FUNCTIONING DIFFICULTIES (N = 954)		BOTH SEVERE & MODERATE-TO-SIGNIFICANT FUNCTIONING DIFFICULTIES (N = 1248)	

Not Documented	394	13.16%	119	15.01%	111	11.64%	164	13.14%

Individual Therapy	780	26.04%	201	25.35%	242	25.37%	337	27.00%

Group Therapy	70	2.34%	40	5.04%	15	1.57%	15	1.20%

Psychoeducation	61	2.04%	28	3.53%	22	2.31%	11	0.88%

Self Help	12	0.40%	7	0.88%	1	0.10%	4	0.32%

MH (Psychosocial)	234	7.81%	65	8.20%	62	6.50%	107	8.57%

MH (Medical/Psychiatric)	86	2.87%	16	2.02%	28	2.94%	42	3.37%

MH (Specialist/Service Package)	73	2.44%	0	0.00%	4	0.42%	69	5.53%

Crisis Intervention	23	0.77%	1	0.13%	6	0.63%	16	1.28%

Case Management	579	19.33%	170	21.44%	186	19.50%	223	17.87%

Online/E-mmh/Helplines	4	0.13%	3	0.38%	1	0.10%	0	0.00%

Hospitalization/ER	8	0.27%	0	0.00%	0	0.00%	8	0.64%

Alcohol or Drug Intervention	31	1.04%	4	0.50%	13	1.36%	14	1.12%

Physical Health	130	4.34%	31	3.91%	33	3.46%	66	5.29%

Family/Carer Intervention	7	0.23%	0	0.00%	3	0.31%	4	0.32%

Peer Support	186	6.21%	66	8.32%	50	5.24%	70	5.61%

Work/School/Primary Needs	68	2.27%	20	2.52%	14	1.47%	34	2.72%

Gender/Sexuality/Sexual Health	11	0.37%	3	0.38%	3	0.31%	5	0.40%

Evaluation/Assessment	38	1.27%	2	0.25%	13	1.36%	23	1.84%

Referral/Navigation	9	0.30%	2	0.25%	4	0.42%	3	0.24%

Other Services	6	0.20%	0	0.00%	1	0.10%	5	0.40%

Support in Initial Assessment	61	2.04%	38	4.79%	17	1.78%	6	0.48%

Single Session + Care Coordination	675	22.54%	151	19.04%	300	31.45%	224	17.95%


*Note*: “Not Documented” does not necessarily mean no services received; it could be missing data or a service not logged in initial service records.

#### By Site

First services offered varied substantially when stratified by site. For example, Individual Therapy was provided to between 6.78% to 80.73% of participants, depending on the site. Similarly, Group Therapy (58.14%), Peer Support (77.97%), Psychoeducation (41.28%), and Single Session/Care Coordination (66.90%) were each offered to a large majority of the service population at one specific site alone, while each of these services was offered to less than 10% of participants across all other sites (Supp Table 7).

#### By Presenting Problem

Crisis intervention (n = 23) was most frequently provided for participants presenting with suicidality or anxiety (each 60.87%). Specialists/specialized care packages were offered more frequently to young people with the following presenting problems: “financial instability” (69.86%), homelessness” (69,86%), “other psychological concerns” (67.12%), “substance use concerns” (58.90%), and “survival situations re: basic needs” (58.90%). Connections to a hospital or emergency room (n = 8) were most frequently provided for young people presenting with substance use concerns (50%), intrusive/obsessive thoughts (50%), or other psychological concerns (50%) (Supp Table 8).

## Discussion

This report describes the problems young people presented with to AOM and the services they received, stratified by a composite measure of clinical severity (CGI) and functioning (SOFAS) assessments at intake. Notably, youth with both moderate-severe clinical severity and poor functioning comprised the most common presentation group, highlighting the prevalence of heightened needs seen in Canadian primary YMH care. This also underscores the vital importance of multidimensional intake assessments in integrated care interventions to accurately identify and respond to the needs of young people who may require more intensive and specialized services [29].

A publication in the larger AOM sample found increased delays for those with moderate-severe presentations [19]. In the current analyses, we report that the highest proportion of young people to receive either 2 or 3+ services on the earliest recorded date were those who presented as neither moderate-severe nor with poor functioning. While this may be due to appropriate referrals to singular services for youth with heightened needs (i.e. a connection to a specialized multicomponent service), it also may be due to limited availability of, or connections to, higher intensity supports across many sites. Young people with both moderate-severe clinical severity and poor functioning also infrequently received a specialist care package and crisis intervention as their first service received, and received case management less frequently than other severity categories. Together, these findings suggest that enhanced primary youth mental health services must make dedicated efforts to adequately and consistently meet the needs of this vulnerable population – either through internal services or referral to higher-intensity care. In future AOM evaluations, it will be critical to explore clinical outcomes by presenting needs in order to assess potential care inequities across sites.

For most young people, the earliest care received following intake entailed one documented service, though the type and number of services received was not documented for a small proportion across severity categories, including those with both moderate-severe presentations and poor functioning. This highlights a group of young people for whom care details are missing. As this group includes a number of young people who present with significant distress, improving service data collection is essential to better understand how AOM sites are supporting them. Future research should also prioritize understanding the factors (e.g. sociodemographic and clinical characteristics, service and/or system insufficiencies) that may be contributing to services not being documented for this group in order to identify any populations that may be disproportionately affected. This could include a range of possibilities, including insufficient training of front-line staff to appreciate emerging or existing mental disorders or that youth with less severe presentations are better able to articulate or assert their needs such that increased services are provided.

Finally, the substantial variation in services received across sites suggests that some young people may not consistently have access to the services they require across site locations. Therefore, it is essential to assess the appropriateness of services offered across sites [19], especially for those presenting with severe and/or complex concerns who may require consistent delivery of more intensive and/or specialized supports, to identify gaps and ensure care provided is aligned with need [30,31]. There is also a need to explore potential barriers to consistent, appropriate service delivery, such as a lack of specialist care, internal workforce training gaps, or service allocation processes that may favour less complex presentations. This also underscores the need to define a minimum core set of interventions available at all sites to set a standard for baseline service delivery, reduce inequities, and support more consistent and appropriate care delivery while allowing for local adaptations.

### Limitations

There are limitations to these analyses. First, providers could select multiple presenting problems, leading to potential variation in how similar presentations were documented between sites and providers. These may not have consistently aligned with youth perceptions. Second, although there were attempts to build a common understanding of what constituted individual services (e.g. case management), these likely varied across sites. These analyses are also limited to *documented* first services received, and service data is often imperfect and limited. These analyses also do not include data for youth who dropped out of care or were referred elsewhere. As such, these data do not likely represent the full services received by young people who presented to AOM sites. Third, the substantial volume of missing data, low cell sizes for certain indicators, and the exclusion of some smaller sites reduces generalizability to the full youth population accessing AOM. Finally, these analyses are limited by their descriptive nature and cannot be generalized to the full population of youth with mental health issues in Canada, nor can we determine the strength of any associations.

## Conclusion

AOM represents progress in YMH systems reform in Canada, successfully bringing mental health services to thousands of young people who might have otherwise gone without appropriate care. While celebrating progress, however, we need to evaluate implementation and identify access inequities to inform continual improvements. Our analysis improves understandings of the needs of young people seeking care in primary YMH interventions, the majority of which presented to AOM sites across Canada with moderate-severe mental health presentations on at least one measure – and many on two. This confirms the need to consistently identify young people with severe, complex, and multidimensional mental health needs in primary care settings and ensure consistent linkages to specialist mental health care via these infrastructures. Our findings also highlight the need to define a core set of interventions offered across integrated youth mental health service sites, including for youth with heightened needs. Future research should assess the appropriateness of services provided in different primary YMH care sites, identify gaps across a full spectrum of need, and work toward standardizing minimum offerings and service definitions in order to ensure equitable, efficient care for all young people.

## Additional File

The additional file for this article can be found as follows:

10.5334/ijic.10147.s1 Supplementary File. Supplemental Data Tables 1 to 8.
