# Supplementary material for: Exploring the Presenting Problems and Services Received by Youth with Diverse Mental Health Presentations in Integrated Youth Mental Health Services Across Canada
Source: Int J Integr Care. 2026 Mar 18;26(1):11. doi: 10.5334/ijic.10147 (PMC13004059; doi:10.5334/ijic.10147)
Supplement: Supplementary File. — Supplemental Data Tables 1 to 8. [file ijic-26-1-10147-s1.pdf]

SUPPLEMENTAL DATA TABLES

Supplemental Table 1: Demographics in the Complete Sample

Site	N	Gender				Mean Age		Sexual Orientation			Ethnic or cultural origins				Housing Insecurity		
		Cis - F	Cis - M	Trans / Other ID	Missing Data	Age	Standard Deviation	Straight	LGB+	MD	White	Visible Minority	Indigenous	MD	Stable	Precarious	MD
**1**	**475**	62.11%	33.89%	4.00%	0.00%	18.05	3.93	23.16%	17.47%	59.37%	30.53%	42.74%	1.68%	25.05%	58.11%	4.21%	37.68%
**2**	**278**	52.52%	32.37%	3.24%	11.87%	17.22	2.78	45.68%	15.11%	39.21%	27.70%	59.35%	0.72%	12.23%	64.03%	2.16%	33.81%
**3**	**681**	36.56%	56.39%	6.90%	0.15%	22.37	2.32	11.16%	8.08%	80.76%	30.25%	16.59%	1.32%	51.84%	24.96%	63.29%	11.75%
**4**	**1288**	60.48%	34.55%	3.96%	1.01%	17.96	2.93	14.21%	8.46%	77.33%	26.16%	3.26%	4.97%	65.61%	25.31%	1.32%	73.37%
**5**	**509**	63.06%	33.01%	2.16%	1.77%	17.9	4.03	20.04%	7.47%	72.50%	0.00%	0.00%	100%	0.00%	30.06%	1.77%	68.17%
**6**	**1562**	39.50%	30.41%	7.23%	22.86%	20.43	2.47	43.92%	33.23%	22.86%	45.39%	14.85%	16.52%	23.24%	67.99%	8.83%	23.18%
**7**	**165**	68.48%	20.00%	9.70%	1.82%	20.19	2.12	54.55%	41.21%	4.24%	21.21%	33.94%	3.03%	41.82%	71.52%	1.21%	27.27%
**8**	**64**	54.69%	40.63%	4.69%	0.00%	16.8	4.15	68.75%	31.25%	0.00%	0.00%	0.00%	100%	0.00%	98.44%	1.56%	0.00%
**9**	**103**	35.92%	46.60%	7.77%	9.71%	16.88	3.02	36.89%	20.39%	42.72%	67.96%	1.94%	6.80%	23.30%	69.90%	4.85%	25.24%
**10**	**80**	41.25%	48.75%	10.00%	0.00%	19.76	2.8	58.75%	40.00%	1.25%	86.25%	6.25%	6.25%	1.25%	73.75%	25.00%	1.25%
**Total**	**5205**	50.43%	35.91%	5.48%	8.18%	19.24	3.45	28.88%	18.96%	52.16%	31.66%	15.72%	17.89%	34.74%	47.98%	12.37%	39.65%

Supplemental Table 2: CGI and SOFAS in the Complete Sample

Site	N	CGI Severity					SOFAS Severity				
		Mild		Severe		% Missing	Mild		Severe		% Missing
1	**475**	94	19.79%	374	78.74%	1.47%	205	43.16%	257	54.11%	2.74%
2	**278**	98	35.25%	145	52.16%	12.59%	116	41.73%	126	45.32%	12.95%
3	**681**	175	25.70%	438	64.32%	9.99%	41	6.02%	529	77.68%	16.30%
4	**1288**	425	33.00%	437	33.93%	33.07%	423	32.84%	420	32.61%	34.55%
5	**509**	73	14.34%	66	12.97%	72.69%	100	19.65%	37	7.27%	73.08%
6	**1562**	524	33.55%	795	50.90%	15.56%	730	46.73%	429	27.46%	25.80%
7	**165**	87	52.73%	55	33.33%	13.94%	107	64.85%	35	21.21%	13.94%
8	**64**	28	43.75%	35	54.69%	1.56%	33	51.56%	31	48.44%	0.00%
9	**103**	65	63.11%	25	24.27%	12.62%	76	73.79%	15	14.56%	11.65%
10	**80**	32	40.00%	44	55.00%	5.00%	30	37.50%	44	55.00%	7.50%
**Total**	**5205**	1654	31.78%	2414	46.63%	21.59%	1861	36.66%	1923	37.29%	26.05%

Supplemental Table 3: Demographics in the Analytic Sample

Site	N	Gender				Mean Age		Sexual Orientation			Ethnic or Cultural Origins				Housing Insecurity		
		Cis - F	Cis - M	Trans / Other ID	Missing Data	Age	Standard Deviation	Straight	LGB+	MD	White	Visible Minority	Indigenous	MD	Stable	Precarious	MD
**1**	**281**	64.06%	31.67%	4.27%	0.00%	18.23	3.85	31.67%	23.13%	45.20%	39.15%	40.57%	2.14%	18.15%	61.92%	4.63%	33.45%
**2**	**192**	58.85%	36.46%	3.65%	1.04%	17.35	2.82	57.29%	16.67%	26.04%	31.25%	66.15%	1.04%	1.56%	76.04%	2.08%	21.88%
**3**	**508**	36.61%	57.28%	5.91%	0.20%	22.25	2.24	12.99%	10.04%	76.97%	31.69%	18.50%	1.77%	48.03%	26.97%	70.08%	2.95%
**4**	**600**	63.00%	32.67%	4.33%	0.00%	17.83	2.66	19.83%	12.00%	68.17%	33.67%	1.67%	6.17%	58.50%	32.83%	1.67%	65.50%
**5**	**109**	59.63%	32.11%	8.26%	0.00%	18.06	3.82	71.56%	28.44%	0.00%	0.00%	0.00%	100%	0.00%	99.08%	0.92%	0.00%
**6**	**1009**	45.49%	34.99%	8.03%	11.50%	20.41	2.45	49.55%	38.95%	11.50%	52.53%	17.34%	18.24%	11.89%	79.19%	8.82%	11.99%
**7**	**137**	69.34%	18.25%	10.95%	1.46%	20.29	2.19	51.82%	44.53%	3.65%	23.36%	35.77%	2.92%	37.96%	75.91%	1.46%	22.63%
**8**	**59**	54.24%	40.68%	5.08%	0.00%	16.64	4.2	67.80%	32.20%	0.00%	0.00%	0.00%	100%	0.00%	98.31%	1.69%	0.00%
**9**	**86**	36.05%	51.16%	9.30%	3.49%	16.74	3.12	40.70%	23.26%	36.05%	74.42%	2.33%	6.98%	16.28%	75.58%	5.81%	18.60%
**10**	**14**	28.57%	64.29%	7.14%	0.00%	20.71	3.29	85.71%	14.29%	0.00%	100%	0.00%	0.00%	0.00%	71.43%	28.57%	0.00%
**Total**	**2995**	51.52%	37.93%	6.41%	4.14%	19.47	3.31	37.40%	24.41%	37.70%	39.17%	19.07%	13.89%	27.88%	60.03%	13.66%	23.77%

Supplemental Table 4: Presenting Problems by Severity

Presenting Problem (Provider Reported)	Total		Severity Measures					
	(N=2995)		Not Severe; Not Moderate-to-Significant Functioning Difficulties (N=793)		Either Severe or Moderate-to-Significant Functioning Difficulties (N=954)		Both Severe & Moderate-to-Significant Functioning Difficulties (N=1248)	
Anxiety/Worry	1279	42.70%	331	41.74%	379	39.73%	569	45.59%
Depression / Sadness	898	29.98%	214	26.99%	273	28.62%	411	32.93%
Stress	887	29.62%	254	32.03%	250	26.21%	383	30.69%
Sleep Issues	675	22.54%	161	20.30%	206	21.59%	308	24.68%
Loneliness / Isolation	541	18.06%	107	13.49%	144	15.09%	290	23.24%
Exposure to Family Relationship Issues	517	17.26%	141	17.78%	128	13.42%	248	19.87%
Financial Instability	504	16.83%	54	6.81%	117	12.26%	333	26.68%
Suicidal Thoughts	476	15.89%	66	8.32%	148	15.51%	262	20.99%
Substance Misuse (Self)	469	15.66%	66	8.32%	99	10.38%	304	24.36%
Difficulties with Concentration	458	15.29%	122	15.38%	131	13.73%	205	16.43%
Academic Difficulties	432	14.42%	152	19.17%	111	11.64%	169	13.54%
Anger	419	13.99%	110	13.87%	116	12.16%	193	15.46%
Social Isolation	404	13.49%	62	7.82%	100	10.48%	242	19.39%
Parental / Youth Conflict	391	13.06%	95	11.98%	109	11.43%	187	14.98%
Panic Attacks	384	12.82%	98	12.36%	120	12.58%	166	13.30%
Self Criticism	335	11.19%	101	12.74%	94	9.85%	140	11.22%
Non-Participation in Occupational	318	10.62%	14	1.77%	77	8.07%	227	18.19%
Homelessness (without stable housing)	285	9.52%	4	0.50%	49	5.14%	232	18.59%
Emotional Abuse (Past or Present)	269	8.98%	43	5.42%	85	8.91%	141	11.30%
Intrusive / Obsessive Thoughts	266	8.88%	42	5.30%	61	6.39%	163	13.06%
Friendship Difficulties	265	8.85%	76	9.58%	75	7.86%	114	9.13%
School / Work Avoidance	264	8.81%	55	6.94%	72	7.55%	137	10.98%
Survival Situation re: Basic Needs	257	8.58%	5	0.63%	45	4.72%	207	16.59%
Thoughts of Harming Self	256	8.55%	35	4.41%	75	7.86%	146	11.70%
Other Psych. Concerns	255	8.51%	23	2.90%	57	5.97%	175	14.02%
Lack of Supportive Adult	244	8.15%	43	5.42%	58	6.08%	143	11.46%
Exposure to Traumatic Event	237	7.91%	46	5.80%	87	9.12%	104	8.33%
Romantic Relationship Difficulties	230	7.68%	57	7.19%	72	7.55%	101	8.09%
Change in Appetite / Weight	227	7.58%	34	4.29%	80	8.39%	113	9.05%
Physical Health Concerns	190	6.34%	42	5.30%	57	5.97%	91	7.29%
Problems with Memory	176	5.88%	38	4.79%	52	5.45%	86	6.89%

Presenting Problem (Provider Reported)	Total		Severity Measures					
	(N=2995)		Not Severe; Not Moderate-to-Significant Functioning Difficulties (N=793)		Either Severe or Moderate-to-Significant Functioning Difficulties (N=954)		Both Severe & Moderate-to-Significant Functioning Difficulties (N=1248)	
Self-Injury / Self Harm	176	5.88%	32	4.04%	44	4.61%	100	8.01%
Bullying (Victim or Perpetrator)	173	5.78%	48	6.05%	44	4.61%	81	6.49%
Sexual Abuse (Part of Present)	169	5.64%	24	3.03%	60	6.29%	85	6.81%
At risk of Being Homeless	164	5.48%	15	1.89%	42	4.40%	107	8.57%
Body Image Issues	157	5.24%	43	5.42%	56	5.87%	58	4.65%
Parental Separation / Divorce	157	5.24%	43	5.42%	46	4.82%	68	5.45%
Physical Abuse (Part of Present)	153	5.11%	21	2.65%	34	3.56%	98	7.85%
Romantic Breakup	148	4.94%	32	4.04%	33	3.46%	83	6.65%
Sig. Decrease/Increase in Energy	143	4.77%	17	2.14%	60	6.29%	66	5.29%
Hearing Voices / Auditory Hallucinations	130	4.34%	9	1.13%	23	2.41%	98	7.85%
Violence / Aggression Towards Others	129	4.31%	20	2.52%	17	1.78%	92	7.37%
Exposure to Family Violence	119	3.97%	27	3.40%	42	4.40%	50	4.01%
Bereavement	110	3.67%	38	4.79%	27	2.83%	45	3.61%
Suicide Attempt	96	3.21%	6	0.76%	26	2.73%	64	5.13%
Disordered Eating	95	3.17%	16	2.02%	38	3.98%	41	3.29%
Grief Resulting from Death	93	3.11%	33	4.16%	29	3.04%	31	2.48%
Behavioural Problems	89	2.97%	21	2.65%	18	1.89%	50	4.01%
Fearing for their Safety	87	2.90%	4	0.50%	15	1.57%	68	5.45%
Criminal Justice Problems	84	2.80%	4	0.50%	12	1.26%	68	5.45%
Exposure to Community Violence	76	2.54%	16	2.02%	33	3.46%	27	2.16%
Other Types of Trauma	73	2.44%	7	0.88%	12	1.26%	54	4.33%
Gender Identity	67	2.24%	7	0.88%	26	2.73%	34	2.72%
Negligence	64	2.14%	11	1.39%	13	1.36%	40	3.21%
Issues with Digestion/Stomach Pains	58	1.94%	13	1.64%	18	1.89%	27	2.16%
Thoughts of Harming Others	56	1.87%	4	0.50%	9	0.94%	43	3.45%
Sexual Orientation	53	1.77%	15	1.89%	19	1.99%	19	1.52%
Elevated Mood / Mania	50	1.67%	3	0.38%	15	1.57%	32	2.56%
Issues re: Cultural Identity	44	1.47%	17	2.14%	19	1.99%	8	0.64%
Substance Misuse (Significant Other)	44	1.47%	10	1.26%	9	0.94%	25	2.00%
Muscle Tension	40	1.34%	11	1.39%	13	1.36%	16	1.28%
Conjugal Violence	32	1.07%	1	0.13%	5	0.52%	26	2.08%
Immigration / Citizenship Status	30	1.00%	8	1.01%	5	0.52%	17	1.36%
Sexual Difficulties	27	0.90%	3	0.38%	11	1.15%	13	1.04%

Presenting Problem (Provider Reported)	Total		Severity Measures					
	(N=2995)		Not Severe; Not Moderate-to-Significant Functioning Difficulties (N=793)		Either Severe or Moderate-to-Significant Functioning Difficulties (N=954)		Both Severe & Moderate-to-Significant Functioning Difficulties (N=1248)	
Discrimination	22	0.73%	6	0.76%	5	0.52%	11	0.88%
Sex Work / Prostitution	19	0.63%	0	0.00%	3	0.31%	16	1.28%
Pregnancy - Carrying to Term	10	0.33%	2	0.25%	5	0.52%	3	0.24%
Pregnancy - Abortion	13	0.43%	3	0.38%	1	0.10%	9	0.72%

Supplemental Table 5: Presenting Problems by Site

	Total (N=2995)	1 (N=281)	2 (N=192)	3 (N=508)	4 (N=600)	5 (N=109)	6 (N=1009)	7 (N=137)	8 (N=59)	9 (N=86)	10 (N=14)
Anxiety/Worry	42.70%	37.37%	32.81%	62.20%	51.17%	84.40%	18.24%	72.99%	61.02%	81.40%	42.86%
Depression / Sadness	29.98%	25.62%	19.79%	35.24%	43.33%	58.72%	17.05%	45.26%	47.46%	25.58%	7.14%
Stress	29.62%	19.22%	30.21%	46.65%	40.67%	81.65%	4.16%	66.42%	32.20%	58.14%	21.43%
Sleep Issues	22.54%	26.69%	18.75%	20.67%	33.50%	53.21%	9.32%	47.45%	38.98%	18.60%	14.29%
Loneliness / Isolation	18.06%	12.10%	18.75%	35.83%	17.17%	36.70%	4.36%	45.26%	30.51%	22.09%	21.43%
Exposure to Family Relationship Issues	17.26%	14.59%	17.19%	28.35%	24.67%	46.79%	2.68%	23.36%	22.03%	30.23%	14.29%
Financial Instability	16.83%	5.69%	8.33%	66.93%	4.00%	9.17%	5.75%	22.63%	10.17%	2.33%	7.14%
Suicidal Thoughts	15.89%	17.08%	14.58%	18.11%	26.67%	36.70%	4.46%	18.25%	42.37%	15.12%	0.00%
Substance Misuse (Self)	15.66%	6.41%	4.17%	47.64%	14.83%	15.60%	6.64%	5.11%	18.64%	10.47%	7.14%
Difficulties with Concentration	15.29%	18.86%	21.35%	13.98%	18.67%	44.95%	4.66%	25.55%	33.90%	31.40%	21.43%
Academic Difficulties	14.42%	12.10%	16.67%	8.27%	17.00%	41.28%	3.47%	65.69%	40.68%	30.23%	14.29%
Anger	13.99%	10.68%	16.67%	13.98%	22.17%	44.95%	4.46%	7.30%	30.51%	33.72%	14.29%
Social Isolation	13.49%	9.25%	8.85%	32.48%	12.67%	29.36%	1.98%	37.23%	5.08%	13.95%	14.29%
Parental / Youth Conflict	13.06%	7.12%	13.02%	21.85%	16.83%	33.03%	4.36%	22.63%	16.95%	12.79%	14.29%
Panic Attacks	12.82%	16.37%	11.46%	8.27%	28.50%	29.36%	2.38%	18.98%	15.25%	13.95%	0.00%
Self Criticism	11.19%	9.25%	15.10%	9.25%	16.67%	51.38%	1.09%	35.77%	18.64%	5.81%	7.14%
Non- Participation in Occupation	10.62%	6.05%	3.65%	39.57%	3.67%	10.09%	5.15%	0.73%	6.78%	1.16%	14.29%
Homelessness (without stable housing)	9.52%	0.00%	0.00%	49.21%	0.67%	0.92%	2.48%	2.92%	1.69%	0.00%	0.00%
Emotional Abuse (Past or Present)	8.98%	9.61%	5.73%	16.34%	11.17%	26.61%	1.68%	15.33%	15.25%	4.65%	7.14%
Intrusive / Obsessive Thoughts	8.88%	2.85%	5.73%	25.39%	10.17%	19.27%	1.19%	9.49%	8.47%	3.49%	21.43%
Friendship Difficulties	8.85%	3.20%	9.38%	8.27%	15.00%	33.03%	1.39%	22.63%	15.25%	11.63%	42.86%
School / Work Avoidance	8.81%	6.41%	13.54%	3.74%	15.83%	33.03%	2.58%	16.79%	27.12%	4.65%	7.14%
Survival Situation re: Basic Needs	8.58%	0.71%	0.00%	48.62%	0.33%	0.00%	0.20%	2.19%	1.69%	0.00%	0.00%
Thoughts of Harming Self	8.55%	4.98%	5.73%	9.84%	16.33%	30.28%	1.09%	12.41%	27.12%	6.98%	0.00%
Other Psych. Concerns	8.51%	5.69%	1.04%	29.13%	0.50%	1.83%	7.23%	2.92%	5.08%	4.65%	0.00%
Lack of Supportive Adult	8.15%	5.34%	4.69%	22.64%	5.67%	16.51%	0.50%	17.52%	28.81%	8.14%	0.00%
Exposure to Traumatic Event	7.91%	7.47%	4.17%	10.43%	7.67%	36.70%	3.47%	12.41%	16.95%	5.81%	14.29%
Romantic Relationship Difficulties	7.68%	3.56%	7.29%	13.98%	8.00%	25.69%	2.78%	15.33%	13.56%	1.16%	7.14%
Change in Appetite / Weight	7.58%	12.46%	3.65%	6.50%	13.83%	20.18%	2.97%	8.03%	5.08%	3.49%	0.00%
Physical Health Concerns	6.34%	6.05%	5.21%	7.28%	7.17%	12.84%	1.68%	27.01%	11.86%	3.49%	35.71%
Problems with Memory	5.88%	6.76%	6.25%	2.95%	9.83%	28.44%	2.08%	5.84%	11.86%	1.16%	21.43%
Self-Injury / Self Harm	5.88%	6.41%	5.21%	6.50%	9.50%	15.60%	1.98%	7.30%	10.17%	5.81%	0.00%
Bullying (Victim or Perpetrator)	5.78%	4.27%	4.17%	7.28%	8.00%	20.18%	0.10%	3.65%	35.59%	19.77%	14.29%
Sexual Abuse (Part of Present)	5.64%	7.12%	1.04%	8.27%	6.50%	17.43%	1.88%	10.22%	18.64%	2.33%	7.14%
At risk of Being Homeless	5.48%	1.07%	0.00%	26.18%	1.17%	1.83%	1.29%	2.92%	3.39%	0.00%	0.00%

Body Image Issues	5.24%	7.12%	2.60%	2.56%	8.17%	24.77%	0.20%	18.25%	18.64%	5.81%	0.00%
Parental Separation / Divorce	5.24%	3.91%	3.13%	6.69%	7.67%	16.51%	0.79%	4.38%	5.08%	27.91%	7.14%
Physical Abuse (Part of Present)	5.11%	5.34%	0.52%	10.04%	6.50%	11.01%	0.99%	7.30%	16.95%	4.65%	7.14%
Romantic Breakup	4.94%	3.56%	3.65%	12.01%	4.33%	9.17%	1.19%	8.03%	13.56%	2.33%	7.14%
Sig. Decrease/Increase in Energy	4.77%	9.96%	1.56%	4.53%	4.00%	11.01%	4.46%	3.65%	3.39%	1.16%	0.00%
Hearing Voices / Auditory Hallucinations	4.34%	2.85%	0.52%	12.80%	2.33%	3.67%	3.17%	0.73%	5.08%	2.33%	0.00%
Violence / Aggression Towards Others	4.31%	4.98%	2.08%	9.06%	5.17%	8.26%	0.79%	0.00%	25.42%	1.16%	7.14%
Exposure to Family Violence	3.97%	2.85%	3.13%	5.51%	3.33%	28.44%	0.40%	5.11%	15.25%	5.81%	7.14%
Bereavement	3.67%	2.49%	3.65%	7.48%	5.00%	4.59%	0.20%	4.38%	8.47%	10.47%	7.14%
Suicide Attempt	3.21%	3.20%	1.04%	4.33%	5.00%	9.17%	0.79%	2.92%	15.25%	2.33%	0.00%
Disordered Eating	3.17%	4.27%	5.73%	1.97%	5.17%	5.50%	1.19%	6.57%	5.08%	1.16%	0.00%
Grief Resulting from Death	3.11%	2.49%	1.56%	3.74%	3.50%	12.84%	0.50%	6.57%	11.86%	9.30%	0.00%
Behavioural Problems	2.97%	3.20%	2.08%	3.74%	4.50%	10.09%	0.00%	2.19%	20.34%	2.33%	14.29%
Fearing for their Safety	2.90%	1.42%	2.08%	12.01%	0.83%	0.00%	0.50%	2.92%	5.08%	1.16%	0.00%
Criminal Justice Problems	2.80%	0.71%	0.52%	10.83%	2.17%	0.92%	0.59%	0.73%	5.08%	2.33%	0.00%
Exposure to Community Violence	2.54%	1.07%	1.56%	1.97%	0.67%	47.71%	0.00%	0.73%	3.39%	0.00%	7.14%
Other Types of Trauma	2.44%	2.85%	0.52%	6.89%	2.00%	0.92%	0.79%	2.19%	6.78%	1.16%	0.00%
Gender Identity	2.24%	1.07%	1.56%	3.74%	2.33%	7.34%	0.79%	7.30%	3.39%	0.00%	0.00%
Negligence	2.14%	4.27%	1.56%	5.91%	1.17%	0.00%	0.20%	2.19%	10.17%	1.16%	0.00%
Issues with Digestion/Stomach Pains	1.94%	2.49%	0.52%	0.98%	3.50%	12.84%	0.30%	3.65%	0.00%	2.33%	0.00%
Thoughts of Harming Others	1.87%	0.71%	0.00%	6.50%	1.17%	3.67%	0.20%	1.46%	10.17%	0.00%	0.00%
Sexual Orientation	1.77%	1.07%	1.04%	1.57%	2.00%	5.50%	0.89%	5.11%	3.39%	4.65%	0.00%
Elevated Mood / Mania	1.67%	1.78%	0.52%	3.54%	2.33%	0.92%	0.79%	0.00%	3.39%	1.16%	0.00%
Issues re: Cultural Identity	1.47%	1.07%	3.13%	0.39%	0.33%	1.83%	0.20%	18.98%	1.69%	0.00%	0.00%
Substance Misuse (Significant Other)	1.47%	2.14%	1.04%	3.54%	1.33%	2.75%	0.20%	0.00%	3.39%	3.49%	0.00%
Muscle Tension	1.34%	0.71%	0.52%	1.38%	1.00%	14.68%	0.00%	3.65%	5.08%	0.00%	0.00%
Conjugal Violence	1.07%	2.14%	0.00%	4.53%	0.17%	0.00%	0.00%	0.73%	0.00%	1.16%	0.00%
Immigration / Citizenship Status	1.00%	0.71%	2.08%	3.15%	0.00%	0.00%	0.10%	5.11%	0.00%	0.00%	0.00%
Sexual Difficulties	0.90%	1.42%	1.04%	2.76%	0.17%	0.00%	0.10%	2.92%	0.00%	1.16%	0.00%
Discrimination	0.73%	0.36%	1.56%	1.57%	0.17%	3.67%	0.00%	1.46%	5.08%	0.00%	0.00%
Sex Work / Prostitution	0.63%	1.07%	0.00%	2.76%	0.00%	0.00%	0.10%	0.00%	1.69%	0.00%	0.00%
Pregnancy - Abortion	0.43%	1.07%	0.00%	0.59%	0.67%	0.00%	0.10%	0.73%	0.00%	1.16%	0.00%
Pregnancy - Carrying to Term	0.33%	0.00%	0.00%	0.98%	0.00%	1.83%	0.20%	0.73%	0.00%	0.00%	0.00%

Supplemental Table 6: Number of Services Received by Site

Services Received	Total (N=2995)	1 (N=281)	2 (N=192)	3 (N=508)	4 (N=600)	5 (N=109)	6 (N=1009)	7 (N=137)	8 (N=59)	9 (N=86)	10 (N=14)
**Not Documented**	13.16%	42.70%	21.35%	10.83%	15.00%	11.93%	2.38%	16.06%	6.78%	22.09%	42.86%
**One**	71.42%	53.38%	43.23%	78.35%	78.33%	32.11%	81.07%	64.96%	71.19%	53.49%	57.14%
**Two**	12.69%	3.20%	30.21%	8.66%	6.67%	18.35%	15.66%	13.87%	18.64%	24.42%	0.00%
**Three+**	2.74%	0.71%	5.21%	2.17%	0.00%	37.61%	0.89%	5.11%	3.38%	0.00%	0.00%

Supplemental Table 7: Service Type by Site

Services Received Type	Total (N=2995)	1 (N=281)	2 (N=192)	3 (N=508)	4 (N=600)	5 (N=109)	6 (N=1009)	7 (N=137)	8 (N=59)	9 (N=86)	10 (N=14)
Not Documented	13.16%	42.70%	21.35%	10.83%	15.00%	11.93%	2.38%	16.06%	6.78%	22.09%	42.86%
Individual Therapy	26.04%	17.79%	39.06%	44.49%	10.67%	80.73%	20.02%	27.74%	6.78%	37.21%	7.14%
Group Therapy	2.34%	1.42%	3.65%	0.20%	0.33%	0.00%	0.30%	1.46%	0.00%	58.14%	7.14%
Psychoeducation	2.04%	1.07%	0.52%	0.20%	0.50%	41.28%	0.00%	5.84%	0.00%	0.00%	0.00%
Self Help	0.40%	0.00%	0.00%	0.00%	0.00%	0.00%	0.59%	2.92%	0.00%	0.00%	14.29%
Psychosocial	7.81%	12.10%	28.65%	9.25%	1.33%	46.79%	1.39%	10.22%	6.78%	5.81%	14.29%
Med / Psychiatric	2.87%	1.78%	6.25%	6.10%	1.50%	0.00%	2.28%	4.38%	0.00%	0.00%	0.00%
Specialist/ Package	2.44%	0.71%	0.00%	13.39%	0.17%	0.00%	0.10%	0.00%	1.69%	0.00%	0.00%
Crisis Intervention	0.77%	0.71%	2.08%	1.18%	0.83%	4.59%	0.00%	0.73%	0.00%	0.00%	0.00%
Case Management	19.30%	13.52%	30.21%	0.98%	47.83%	0.00%	17.34%	10.22%	0.00%	0.00%	7.14%
Online/E-mh/Helplines	0.13%	0.00%	0.00%	0.00%	0.00%	0.00%	0.10%	2.19%	0.00%	0.00%	0.00%
Hospitalization / ER	0.27%	0.00%	0.00%	1.18%	0.17%	0.00%	0.10%	0.00%	0.00%	0.00%	0.00%
Alcohol/Drug Int	1.04%	0.00%	0.00%	1.77%	0.33%	2.75%	1.59%	0.00%	1.69%	0.00%	0.00%
Physical Health	4.34%	0.36%	0.00%	17.91%	0.33%	1.83%	0.99%	15.33%	3.39%	0.00%	7.14%
Family / Carer Int	0.23%	0.00%	1.04%	0.00%	0.17%	0.00%	0.10%	0.73%	1.69%	1.16%	0.00%
Peer Support	6.21%	0.00%	0.52%	0.00%	21.33%	0.92%	0.50%	3.65%	77.97%	0.00%	0.00%
Work/ School/ Needs	2.27%	0.00%	1.04%	3.74%	1.33%	0.92%	0.69%	15.33%	16.95%	0.00%	0.00%
Gender/Sexuality	0.37%	0.00%	0.00%	1.18%	0.00%	2.75%	0.10%	0.73%	0.00%	0.00%	0.00%
Evaluation/Assessment	1.27%	11.03%	2.60%	0.39%	0.00%	0.00%	0.00%	0.00%	0.00%	0.00%	0.00%
Referral / Navigation	0.30%	0.71%	0.52%	0.39%	0.17%	0.00%	0.20%	0.00%	1.69%	0.00%	0.00%
Other Services	0.20%	0.36%	0.00%	0.39%	0.17%	0.00%	0.20%	0.00%	0.00%	0.00%	0.00%
Support in Assessment	2.04%	0.36%	3.13%	0.00%	4.17%	0.92%	1.59%	8.03%	1.69%	0.00%	0.00%
Single Session + Care Coordination	22.54%	0.00%	0.00%	0.00%	0.00%	0.00%	66.90%	0.00%	0.00%	0.00%	0.00%

Supplemental Table 8: Presenting Problem by Service Type (%)

	Total	Not-Doc	Ind - Ther	Group Ther	Psy Edu	Self Help	Psy - Soc	Med Psych	Spec/ Pack	Crisis Int	Case Mgmt	E-MH	Hosp / ER	Alc / Drug	Phys Health	Fam / CG	Peer Supp	Work / Edu	Gend/ Sex	Eval / Asmt	Ref / Nav	Other	Single Sess	SS / Coord
***N*=**	**2995**	**394**	**780**	**70**	**61**	**12**	**234**	**86**	**73**	**23**	**578**	**4**	**8**	**31**	**130**	**7**	**186**	**68**	**11**	**38**	**9**	**6**	**61**	**675**
Anxiety	54.29	52.03	63.59	75.71	81.97	41.67	51.28	55.81	43.84	60.87	39.62	75.00	37.50	45.16	66.92	71.43	72.04	60.29	81.82	36.84	66.67	66.67	55.74	1.04
Depression	38.51	33.50	48.85	22.86	57.38	16.67	30.34	39.53	15.07	52.17	32.01	50.00	25.00	38.71	47.69	57.14	60.22	32.35	54.55	23.68	44.44	33.33	31.15	1.48
Stress	37.90	34.26	43.08	57.14	81.97	33.33	43.59	34.88	32.88	47.83	23.88	75.00	25.00	32.26	49.23	42.86	53.23	51.47	54.55	21.05	33.33	0.00	36.07	0.15
Sleep Issues	29.01	30.20	32.18	20.00	50.82	50.00	24.79	29.07	15.07	47.83	24.57	25.00	25.00	25.81	35.38	42.86	43.01	26.47	36.36	23.68	33.33	33.33	24.59	0.44
Loneliness	22.97	17.01	26.67	25.71	37.70	33.33	26.50	23.26	42.47	34.78	14.36	0.00	12.50	22.58	33.08	57.14	32.26	32.35	18.18	13.16	22.22	50.00	14.75	0.00
Family	21.88	19.29	25.64	24.29	45.90	33.33	24.36	8.14	32.88	21.74	14.36	0.00	37.50	19.35	23.08	28.57	33.33	26.47	18.18	18.42	11.11	33.33	22.95	0.30
Financial	20.54	14.97	26.03	8.57	13.11	8.33	22.65	29.07	69.86	17.39	8.30	0.00	25.00	41.94	57.69	14.29	7.53	35.29	45.45	15.79	11.11	50.00	6.56	0.59
Suicidality	19.99	18.78	21.54	14.29	32.79	16.67	19.23	20.93	19.18	60.87	14.36	0.00	12.50	35.48	24.62	14.29	37.63	17.65	36.36	10.53	33.33	0.00	8.20	0.44
Concentration	19.60	21.83	19.87	30.00	37.70	25.00	19.66	29.07	31.51	34.78	14.53	0.00	12.50	3.23	16.15	71.43	22.58	19.12	18.18	21.05	55.56	0.00	13.11	0.30
Substance (S)	19.21	17.26	19.87	8.57	16.39	8.33	17.09	27.91	58.90	30.43	11.07	0.00	50.00	80.65	39.23	14.29	22.04	11.76	36.36	10.53	22.22	33.33	9.84	0.59
Academic	18.82	19.04	19.74	35.71	45.90	25.00	24.79	12.79	5.48	13.04	10.55	50.00	0.00	12.90	20.00	42.86	30.65	30.88	18.18	10.53	11.11	0.00	26.23	0.15
Anger	18.36	18.78	20.77	34.29	34.43	8.33	19.23	10.47	19.18	26.09	15.40	0.00	12.50	22.58	13.08	42.86	29.03	8.82	9.09	7.89	11.11	16.67	8.20	0.30
P. Conflict	17.03	12.44	20.64	14.29	29.51	8.33	17.95	20.93	20.55	17.39	12.46	0.00	37.50	16.13	18.46	14.29	23.12	27.94	27.27	0.00	22.22	16.67	16.39	0.74
Isolation	16.95	11.93	21.54	18.57	32.79	33.33	23.08	20.93	39.73	17.39	7.27	50.00	12.50	19.35	24.62	0.00	16.67	19.12	18.18	10.53	33.33	16.67	11.48	0.30
Panic	15.94	18.02	15.38	18.57	32.79	8.33	12.82	20.93	2.74	26.09	12.28	25.00	0.00	3.23	18.46	0.00	32.26	17.65	9.09	7.89	44.44	0.00	21.31	0.44
Self Crit	15.08	14.21	17.95	10.00	45.90	33.33	19.23	15.12	4.11	26.09	7.09	0.00	0.00	9.68	17.69	14.29	23.66	20.59	27.27	7.89	22.22	0.00	18.03	0.15
Non-Part	12.93	8.88	15.00	2.86	8.20	8.33	12.82	22.09	54.79	21.74	6.06	0.00	25.00	35.48	31.54	0.00	6.45	22.06	0.00	7.89	22.22	16.67	6.56	0.59
Avoidance	12.05	8.88	12.95	4.29	32.79	0.00	14.53	13.95	6.85	30.43	10.38	50.00	0.00	3.23	7.69	14.29	21.51	20.59	9.09	5.26	0.00	0.00	14.75	0.15
Homeless	11.66	8.38	13.85	0.00	1.64	0.00	12.82	25.58	69.86	17.39	3.11	0.00	25.00	22.58	30.77	14.29	0.54	29.41	18.18	5.26	0.00	33.33	0.00	0.30
Friendship	11.59	11.42	12.69	14.29	37.70	0.00	17.52	4.65	6.85	17.39	7.79	50.00	12.50	9.68	6.15	28.57	17.20	17.65	18.18	0.00	0.00	16.67	8.20	0.00
Emo.Abuse	11.57	10.41	14.23	4.29	24.59	0.00	14.10	15.12	10.96	13.04	6.92	0.00	0.00	6.45	16.15	0.00	13.98	19.12	18.18	10.53	22.22	33.33	6.56	0.15
Other Psyc	11.27	5.58	12.95	5.71	1.64	16.67	10.68	17.44	67.12	13.04	9.52	0.00	50.00	19.35	14.62	14.29	2.69	5.88	27.27	10.53	22.22	33.33	6.56	0.44
Int/Obs	11.04	7.36	11.79	7.14	14.75	16.67	13.25	26.74	39.73	13.04	4.50	25.00	50.00	3.23	21.54	28.57	14.52	10.29	9.09	2.63	22.22	16.67	4.92	0.00
Self Harm-T	10.81	10.15	11.28	10.00	24.59	8.33	9.40	13.95	5.48	26.09	6.23	0.00	12.50	16.13	16.92	14.29	22.04	13.24	9.09	7.89	33.33	0.00	6.56	0.00
Trauma	10.76	9.39	12.44	10.00	40.98	0.00	15.38	8.14	4.11	13.04	6.40	0.00	0.00	19.35	11.54	0.00	14.52	14.71	27.27	5.26	11.11	0.00	4.92	0.15

Basic Needs	10.08	6.09	12.95	1.43	0.00	0.00	12.39	20.93	58.90	8.70	0.69	0.00	25.00	12.90	36.15	0.00	1.08	17.65	36.36	5.26	11.11	33.33	0.00	0.15
Lack Adult	10.07	8.12	12.82	8.57	22.95	8.33	11.54	6.98	19.18	13.04	3.29	0.00	25.00	9.68	16.92	0.00	16.67	16.18	18.18	2.63	22.22	0.00	3.28	0.15
RomanticD	9.95	8.38	15.51	1.43	21.31	16.67	11.11	8.14	1.37	8.70	5.36	0.00	12.50	9.68	12.31	0.00	12.37	10.29	9.09	5.26	11.11	0.00	6.56	0.15
Appetite	9.62	10.15	10.00	2.86	14.75	0.00	6.84	12.79	5.48	30.43	9.17	0.00	0.00	9.68	9.23	0.00	13.98	7.35	18.18	18.42	11.11	0.00	14.75	0.15
Physical	8.52	10.66	7.82	7.14	26.23	0.00	10.26	8.14	9.59	13.04	4.84	0.00	0.00	3.23	14.62	28.57	10.22	14.71	9.09	2.63	22.22	0.00	4.92	0.30
Memory	8.09	9.64	7.69	4.29	21.31	8.33	10.68	10.47	6.85	8.70	6.92	0.00	12.50	6.45	4.62	28.57	10.75	5.88	27.27	7.89	22.22	0.00	1.64	0.00
Self Harm	7.67	7.61	8.59	7.14	14.75	0.00	7.26	16.28	1.37	8.70	4.84	0.00	0.00	0.00	13.08	28.57	11.29	8.82	9.09	10.53	0.00	16.67	4.92	0.00
Bullying	7.31	6.85	6.67	14.29	13.11	0.00	9.83	5.81	8.22	13.04	3.63	0.00	0.00	6.45	7.69	14.29	17.20	13.24	18.18	2.63	11.11	0.00	6.56	0.00
Body Image	7.19	5.58	6.41	2.86	31.15	8.33	10.26	5.81	1.37	4.35	4.50	25.00	0.00	3.23	10.77	14.29	13.44	7.35	36.36	15.79	11.11	0.00	4.92	0.15
Sexual Abuse	7.13	6.09	7.69	5.71	11.48	8.33	8.55	9.30	9.59	4.35	6.06	0.00	0.00	6.45	6.92	0.00	8.06	11.76	18.18	10.53	0.00	0.00	3.28	0.44
P. Separation	6.70	3.81	8.33	20.00	14.75	8.33	6.84	10.47	4.11	0.00	4.33	0.00	0.00	0.00	7.69	42.86	10.22	7.35	0.00	2.63	11.11	0.00	3.28	0.15
Phy Abuse	6.53	5.33	6.79	2.86	11.48	0.00	8.12	10.47	4.11	8.70	3.98	0.00	0.00	6.45	10.00	0.00	11.83	16.18	9.09	5.26	11.11	16.67	1.64	0.15
Homeless (R)	6.49	4.31	11.15	1.43	1.64	0.00	3.85	5.81	12.33	4.35	2.60	0.00	12.50	9.68	21.54	0.00	2.69	8.82	18.18	2.63	11.11	0.00	0.00	0.15
Energy	6.26	5.84	7.82	0.00	9.84	16.67	3.85	6.98	1.37	4.35	7.44	0.00	0.00	12.90	4.62	28.57	4.84	0.00	0.00	10.53	0.00	0.00	9.84	0.44
Breakup	5.83	4.57	8.59	1.43	3.28	0.00	5.56	5.81	6.85	4.35	2.42	0.00	0.00	9.68	6.15	14.29	9.14	10.29	36.36	5.26	11.11	16.67	3.28	0.15
F. Violence	5.62	5.84	6.54	5.71	26.23	0.00	10.26	1.16	9.59	4.35	2.25	0.00	0.00	9.68	3.08	0.00	4.84	10.29	9.09	2.63	11.11	0.00	1.64	0.00
Hallucination	5.58	3.55	4.23	7.14	1.64	0.00	6.41	6.98	52.05	8.70	4.15	0.00	37.50	3.23	4.62	0.00	4.30	1.47	0.00	5.26	11.11	33.33	4.92	0.00
Violence	5.26	4.06	4.10	1.43	4.92	8.33	5.56	9.30	23.29	8.70	4.15	0.00	12.50	6.45	5.38	14.29	10.22	8.82	0.00	2.63	0.00	16.67	1.64	0.00
C. Violence	5.16	1.27	6.79	0.00	45.90	0.00	16.24	0.00	1.37	17.39	1.04	0.00	0.00	9.68	3.08	0.00	2.15	2.94	27.27	2.63	0.00	0.00	1.64	0.00
Suicide (A)	4.38	3.81	5.26	2.86	9.84	0.00	5.13	5.81	1.37	8.70	2.60	0.00	25.00	3.23	4.62	0.00	8.60	2.94	9.09	2.63	11.11	0.00	1.64	0.00
Bereavement	4.36	5.33	5.64	10.00	6.56	0.00	4.70	1.16	1.37	0.00	1.73	0.00	0.00	0.00	10.00	0.00	7.53	0.00	0.00	2.63	0.00	0.00	4.92	0.00
Dis Eating	4.06	4.31	4.10	1.43	3.28	0.00	3.42	3.49	5.48	8.70	3.81	0.00	0.00	0.00	5.38	0.00	6.99	5.88	9.09	5.26	0.00	0.00	3.28	0.15
Grief	4.03	4.06	4.23	7.14	14.75	0.00	5.98	3.49	0.00	8.70	2.60	0.00	0.00	0.00	6.92	0.00	5.38	2.94	0.00	2.63	0.00	0.00	1.64	0.00
B Problems	3.77	5.33	2.31	4.29	6.56	0.00	4.70	4.65	9.59	8.70	2.25	0.00	0.00	3.23	3.08	28.57	6.99	4.41	0.00	2.63	0.00	0.00	8.20	0.00
Fear Safety	3.58	2.79	2.95	1.43	1.64	0.00	2.56	2.33	36.99	4.35	1.90	0.00	12.50	0.00	6.92	0.00	1.61	7.35	0.00	5.26	11.11	16.67	1.64	0.00
Justice	3.16	2.79	2.82	1.43	0.00	0.00	2.99	8.14	20.55	0.00	1.73	0.00	0.00	9.68	7.69	0.00	2.69	2.94	0.00	0.00	0.00	0.00	0.00	0.15
Gender ID	3.08	1.27	3.59	1.43	6.56	8.33	4.70	6.98	2.74	8.70	1.21	0.00	0.00	0.00	4.62	14.29	3.23	4.41	63.64	2.63	0.00	0.00	0.00	0.00
Oth Trauma	2.99	4.06	3.33	2.86	1.64	0.00	2.56	4.65	4.11	4.35	2.60	0.00	12.50	3.23	2.31	0.00	3.23	4.41	0.00	2.63	0.00	0.00	0.00	0.00
Digestion	2.86	3.30	2.56	1.43	18.03	0.00	4.70	1.16	0.00	13.04	1.73	0.00	0.00	0.00	1.54	14.29	3.23	4.41	0.00	5.26	0.00	0.00	1.64	0.00
Negligence	2.45	2.28	3.72	0.00	1.64	0.00	1.28	0.00	2.74	0.00	1.38	0.00	0.00	0.00	3.85	0.00	3.76	8.82	0.00	7.89	0.00	0.00	0.00	0.00

Sex Orient	2.32	1.78	3.21	1.43	4.92	8.33	2.99	5.81	0.00	4.35	1.21	0.00	0.00	0.00	2.31	0.00	2.69	1.47	18.18	0.00	0.00	0.00	1.64	0.00
Harm Others	2.30	2.03	1.28	0.00	1.64	0.00	2.14	6.98	15.07	4.35	1.21	0.00	12.50	3.23	5.38	0.00	3.23	2.94	0.00	2.63	11.11	0.00	0.00	0.00
Musc Tension	2.26	0.51	3.08	0.00	13.11	0.00	5.56	0.00	1.37	8.70	0.87	0.00	0.00	3.23	1.54	0.00	2.15	2.94	9.09	2.63	11.11	0.00	0.00	0.00
Mania	2.10	1.78	1.28	0.00	0.00	0.00	1.71	2.33	15.07	8.70	1.73	0.00	12.50	9.68	3.08	0.00	2.15	1.47	0.00	2.63	11.11	0.00	0.00	0.15
Substance-O	1.89	1.27	2.05	2.86	4.92	16.67	2.99	0.00	0.00	0.00	0.87	0.00	0.00	0.00	4.62	0.00	1.61	5.88	0.00	2.63	0.00	0.00	3.28	0.00
Conj Violence	1.85	1.78	2.05	2.86	6.56	0.00	2.56	1.16	4.11	4.35	1.21	0.00	0.00	0.00	1.54	0.00	2.69	0.00	0.00	2.63	0.00	0.00	0.00	0.00
Cultural ID	1.18	1.27	1.54	0.00	0.00	0.00	2.14	1.16	2.74	4.35	0.17	0.00	0.00	3.23	2.31	14.29	0.00	2.94	0.00	2.63	0.00	0.00	0.00	0.00
Imm / Citizen	1.18	0.25	1.15	0.00	0.00	8.33	1.71	2.33	5.48	4.35	0.69	0.00	0.00	0.00	3.85	0.00	0.00	1.47	0.00	2.63	0.00	0.00	3.28	0.00
Sex.Difficulties	1.01	1.02	1.28	1.43	1.64	0.00	2.14	1.16	1.37	0.00	0.17	0.00	0.00	0.00	3.08	0.00	0.54	0.00	0.00	0.00	0.00	0.00	0.00	0.15
Discrimination	0.98	0.25	1.15	0.00	3.28	0.00	2.56	0.00	2.74	0.00	0.17	0.00	0.00	0.00	1.54	14.29	1.08	1.47	9.09	0.00	0.00	0.00	1.64	0.00
Prostitution	0.91	0.25	0.90	0.00	0.00	0.00	1.71	2.33	2.74	4.35	0.52	0.00	0.00	0.00	2.31	0.00	0.00	2.94	9.09	2.63	0.00	0.00	0.00	0.00
Pregnancy	0.57	0.25	0.64	0.00	3.28	0.00	0.85	1.16	0.00	0.00	0.17	0.00	0.00	3.23	2.31	0.00	0.00	0.00	9.09	0.00	0.00	0.00	0.00	0.00
Abortion	0.44	0.51	0.38	0.00	0.00	0.00	0.00	1.16	1.37	0.00	0.35	0.00	0.00	0.00	0.00	14.29	0.54	0.00	9.09	2.63	0.00	0.00	0.00	0.00
